# Antidepressant prescribing in Irish children: secular trends and international comparison in the context of a safety warning

**DOI:** 10.1186/s12887-015-0436-2

**Published:** 2015-09-11

**Authors:** K. O’Sullivan, F. Boland, U. Reulbach, N. Motterlini, D. Kelly, K. Bennett, T. Fahey

**Affiliations:** HRB Centre for Primary Care Research, Division of Population Health Sciences, Royal College of Surgeons in Ireland, 123 St Stephens Green, Dublin 2, Ireland; Department of Public Health and Primary Care, Trinity College Centre for Health Sciences, Trinity College Dublin, Dublin 2, Ireland; Department of Pharmacology and Therapeutics, Trinity Centre for Health Sciences, St James’s Hospital, Dublin 8, Ireland

**Keywords:** Children, Anti-depressants, Paediatric prescribing, Safety warning

## Abstract

**Background:**

In 2003, the Irish Medicines Board (IMB) warned against the treatment of childhood depression with selective serotonin reuptake inhibitors (SSRIs) due to increased risk of suicide. This study examined the effect of this warning on the prevalence of anti-depressants in Irish children and compared age and gender trends and international comparisons of prescription rates.

**Methods:**

A retrospective cohort study of the Irish Health Service Executive (HSE) pharmacy claims database for the General Medical Services (GMS) scheme for dispensed medication. Data were obtained for 2002–2011 for those aged ≤15 years. Prevalence of anti-depressants per 1000 eligible population, along with 95 % confidence intervals, were calculated. A negative binomial regression analysis was used to investigate trends and compare rates across years, sex and age groups (0–4, 5–11, 12–15 years). International prescribing data were retrieved from the literature.

**Results:**

The prevalence of anti-depressants decreased from 4.74/1000 population (95 % CI: 4.47-5.01) in 2002 to 2.61/1000 population (95 % CI: 2.43-2.80) in 2008. SSRI rates decreased from 2002 to 2008. Prescription rates for contra-indicated SSRIs paroxetine, sertraline and citralopram decreased significantly from 2002 to 2005, and, apart from paroxetine, only small fluctuations were seen from 2005 onwards. Fluoxetine was the most frequently prescribed anti-depressant and rates increased between 2002 and 2011. Anti-depressant rates were higher for younger boys and older girls. The Irish prevalence was lower than the US, similar to the U.K. and higher than Germany and Denmark.

**Conclusions:**

The direction and timing of these trends suggest that medical practitioners followed the IMB advice.

## Background

Depression is common in young people and contributes to a variety of negative outcomes such as poor academic attainment, difficulty in peer and family relations, and increased risk of suicide [1]. Major depressive disorder has a lifetime prevalence of 20.7 % in adults [1, 2]; and affects up to 10 % of children [3]. Common symptoms of depression in childhood include low mood, loss of interest in once enjoyed activities, psychosomatic symptoms and in severe cases thoughts of suicide [1]. Depression in childhood, if left untreated, is likely to continue into adulthood and over time becomes increasingly difficult to treat [4]. Childhood depression can last for several months, is recurrent and is twice as likely to be observed in teenage girls as teenage boys [5]. A higher rate of depression in teenage girls has been associated with hormonal changes related to puberty rather than age related development [5].

Anti-depressants are often used to treat depression, anxiety and other disorders in children and adolescents [6]. The late 1990’s saw a steady increase in the use of anti-depressants in children, fuelled primarily by the rise in popularity of selective serotonin reuptake inhibitors (SSRIs) [7–9]. This rise was influenced by two factors; early studies showing their effectiveness in treating adult depression and drug trials that showed the ineffectiveness of tryciclic anti-depressants in the treatment of childhood depression [3]. Further support for their use in childhood depression and anxiety came from early randomised controlled trials (RCT) which showed high levels of SSRI efficacy in comparison to placebo [7, 10, 11]. However, reviews of SSRI safety and efficacy in the treatment of childhood depression later revealed they were more harmful than what had been originally reported [12, 13].

In 2003, the Food and Drug Administration (FDA) requested that GlaxoSmithKline (GSK) provide the results of all drug trials that had examined the efficacy of SSRIs in the treatment of Major Depressive Disorder (MDD) in children. This request followed the airing of a documentary which highlighted the side effects of Seroxat (paroxetine) and the suppression of data reporting these side effects by the pharmaceutical industry. In May 2003, the GSK report revealed an association with paroxetine and suicidal behaviour in children. Following this, and other reports the FDA published an advisory paper highlighting the increased risk of suicidal behaviour in children being treated with SSRIs. Later that year, the Medicines and Health Regulatory Agency (MHRA) issued a recommendation to withdraw the use of all SSRIs in children with MDD, except for fluoxetine [12], a move which was endorsed by the Irish Medical Board (IMB). In late 2004, the FDA required that manufacturers add a black box warning to all SSRIs including the risk of suicidal behaviours [13]. The SSRIs paroxetine, sertraline and citralopram were contraindicated in children for the treatment of depression following these warnings. Since 2004 these warnings have been extended, the FDA increased the age bracket from 18 to 24 in 2007 and the IMB adapted the SSRI warning up to the age of 25 in 2008 (see Table 1) [14].

**Table 1:** Summary of the history of SSRI warnings across countries and agencies

Year, Month	Country	Agency	Action
2003, May	-	GlaxoSmithKline	Reported to FDA increased suicidal behaviour associated with paroxetine
2003, June	U.K.	MHRA^a^	Paroxetine contraindicated in <18 s
2003, September	U.K.	MHRA	Venflaxine contraindicated in <18 s
2003, October	U.S.	FDA^b^	Advice paper published stating preliminary data suggests increased suicidal behaviour associated with SSRIs
2003, December	U.K.	MHRA	Contraindicate all SSRIs in <18 s apart from Prozac (fluoxetine)
2003, December	Ireland	IMB^c^	Endorses MHRA warning for Ireland
2004, February	U.S.	FDA	Commissioned advisory committee
2004, October	U.S.	FDA	Issued black box warning relating to all anti-depressants <18 s
2005, August	Europe	EMEA^d^	Issued warning against SSRIs in <18 s
2005, November	Ireland	IMB	Reissued warning and updated label guidelines
2007, May	U.S.	FDA	Increased age of risk of SSRIs to 24 years
2008, September	Ireland	IMB	Warnings adapted up to age 25

^a^Medicines & Health Regulatory Agency ^b^Food & Drug Administration ^c^ Irish Medicines Board ^d^European Medicines Agency

Increased rates in psychotropic drug prescribing in children have been reported in recent times [15–19], and anti-depressants are often the most frequently prescribed [15–17]. While studies have shown that prevalence of anti-depressants declined immediately following the introduction of the FDA boxed warnings [6, 20], there is evidence to suggest that this decline was not sustained and that the prevalence of paediatric anti-depressant prescribing is on the rise [21]. Furthermore, there is wide variability across countries in the use of anti-depressant medication for children. For example, in 2000 in the US, the prevalence was 15 times greater than that of Germany, and the rate in Germany was 9 times greater than that in Denmark [9]. These differences are thought to be due to several factors; including differences in diagnostic criteria, treatment guidelines, drug regulations and healthcare systems [9].

The aims of the current study are (i) to examine whether the introduction of the IMB warnings was associated with a reduction in overall and specific prevalence of anti-depressants in Irish children, and (ii) to establish whether the effect of this warning was maintained. Age and gender trends were also considered, and additionally, the prevalence of anti-depressants in children in Ireland was compared to international studies.

## Methods

### Study population and study design

Data was obtained from the Irish General Medical Services (GMS) scheme pharmacy claims database from the Health Service Executive (HSE) – Primary Care Reimbursement Services (PCRS). The database contains basic demographic information and details of dispensed medications (coded using WHO’s [20] Anatomical Therapeutic Chemical (ATC) classification) for each individual on the GMS scheme. The scheme is means tested and provides free health services to those who are unable to afford them. It represents approximately 28 % of Irish children but over-represents socially deprived populations. Data is freely available but with the necessary confidentiality agreements. Permission was given by the data controller to use the GMS dataset if anonymised and analysed at group level. Therefore, it was unnecessary to seek specific ethical approval for this study.

All anti-depressant medication prescriptions (N06A; classified according to the ATC classification system) were extracted from the GMS database for children aged 0–15 years inclusive for the years 2002–2011.

### Data analysis

The yearly prevalence of anti-depressants per 1000 GMS population and associated 95 % confidence intervals for children aged 0–15 years were calculated as a proportion of all eligible children (0–15 years) entitled to free health services, as identified from the annual reports produced by the PCRS. The prevalence per 1000 GMS eligible population and associated 95 % confidence intervals (CIs) were also calculated across years (2002–2011), age groups (0–4 years, 5–11 years and 12–15 years) and gender.

A negative binomial regression model was used to determine trends in prevalence. The log of the GMS population was used as the offset term and year, age group, gender and all possible interactions between these variables were included as fixed effects in the model. The Bonferroni method was used to control the overall Type I error rate in making multiple comparisons of means and *p*-values <0.05 were deemed significant.

Data analyses was performed using Stata version 11 (StataCorp, College Station, Tx, USA) and SAS version 9.3 (SAS Institute Inc. Cary, NC, USA).

### Comparison studies

Comparison studies, examining the prevalence of anti-depressants in paediatric populations, were identified from a search of published literature from 1995 – 2013. Articles were included if they reported the prevalence of anti-depressants in a community setting and provided overall prevalence. Studies which reported overall percentage prevalence were transformed to per 1000 population to facilitate comparison.

## Results

### Population sample

During the study period, January 2002 to December 2011, the number of children ≤15 years in Ireland, as identified from the HSE-PCRS pharmacy database, ranged between 188,833 and 311,579. On average, 51 % of the study population were male and 49 % were female.

### Prescribing trends

Table 2 shows the prevalence of anti-depressants for 2002–2011. In 2002, 4.74/1000 population (95 % CI: 4.47-5.01) received at least one anti-depressant medication prescription and this decreased yearly to 2.61/1000 population (95 % CI: 2.43-2.80) in 2008. Between 2008 and 2011 the prevalence fluctuated slightly.

**Table 2:** Prevalence and 95 % confidence intervals of anti-depressants to children aged 0–15 years from 2002–2011

Year	Prevalence per 1,000 GMS population (95 % confidence interval)
2002	4.74 (4.47–5.01)
2003	4.33 (4.07–4.59)
2004	3.86 (3.62–4.11)
2005	3.51 (3.27–3.74)
2006	3.09 (2.87–3.30)
2007	2.72 (2.52–2.91)
2008	2.61 (2.43–2.80)
2009	2.71 (2.54–2.89)
2010	2.63 (2.47–2.80)
2011	2.86 (2.69–3.03)

**Fig. 1:**
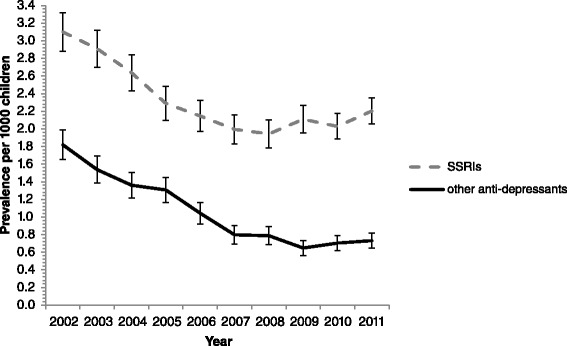
Prevalence of SSRIs (N06AB) vs non-SSRIs (N06AA, N06AF, N06AG, N06AX) per 1000 GMS population aged 0–15 years old from 2002 to 2011

During the study period the prevalence of SSRIs was much higher than non-SSRIs (Fig. 1). There were no significant differences in the prevalence of SSRIs between 2002 and 2003. However, a significant decrease was seen between 2003 and 2004, and 2003 and 2005; the period directly following the IMB warning. Since 2005, the prevalence of SSRIs has remained relatively stable.

**Fig. 2:**
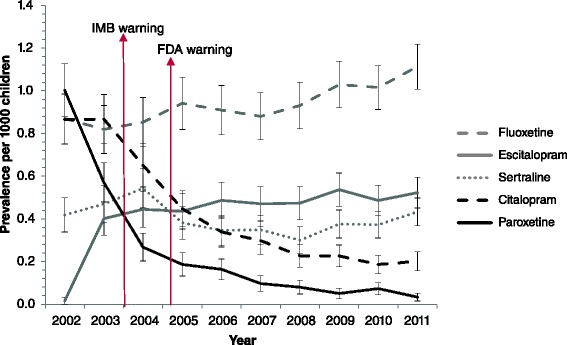
Prevalence of SSRIs per 1000 GMS population aged 0–15 years old from 2002 to 2011 (including when warnings were introduced)

Fluoxetine was the most frequently prescribed anti-depressant (Fig. 2), and although no significant differences were seen, the prevalence of fluoxetine increased between 2002 and 2011. Over the study period a decrease in the prevalence of contra-indicated anti-depressants was seen. The prevalence of paroxetine decreased significantly from 1.00/1000 population (95 % CI: 0.88-1.13) in 2002 to 0.03/1000 population (95 % CI: 0.02-0.05) in 2011. Significant decreases were observed yearly from 2002 through to 2007. From 2007 onwards, the prevalence stabilised. The prevalence of citalopram also decreased significantly over the study period from 0.87/1000 population (95 % CI: 0.75-0.98) in 2002 to 0.19/1000 population (95 % CI: 0.14-0.23) in 2011. The prevalence of sertraline and escitalopram, after its introduction to the market in 2002, fluctuated over the study period (Fig. 2). For sertraline, significant decreases between 2004 and 2006, and 2004 and 2007 were observed.

### Gender and Age

**Fig. 3:**
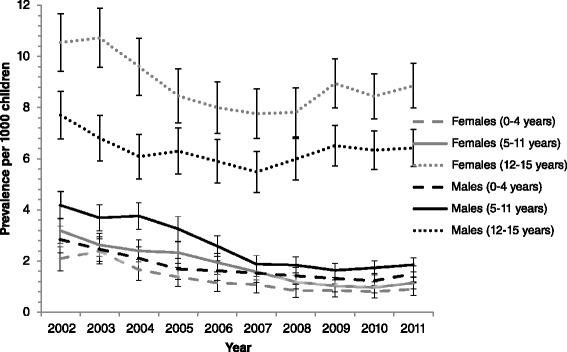
Trends in the prevalence of anti-depressants for 2002–2011, classified by gender and age group

Figure 3 shows the prevalence of anti-depressants for all years for males and females and all age groups (0–4 years, 5–11 years and 12–15 years). The fixed interactions year × gender × age group (*p =* 0.93) and year × gender (*p =* 0.35) were not significant while the interactions age group × year (*p <* 0.0001) and age group × gender (*p <* 0.0001) were significant. This means that the effect of age group on the prevalence of anti-depressants differed over years and differed for males and females also.

The least square means and *t*-tests for the difference in prevalence of anti-depressants between age groups for gender and years showed that significant differences existed between males and females for all age groups whereby males had higher prevalences at 0–4, and 5–11 age groups. Females had higher rates in the 12–15 year age group. Additionally, for all years, significantly higher prevalences were seen for the 12–15 year age group compared to the 0–4 year, and the 5–11 year groups. Overall significantly higher prevalences were seen for the 5–11 year age group compared to the 0–4 year group for 2002, 2004, 2005 and 2006 only.

### Comparison studies

Studies examining the overall prevalence and incidence of anti-depressants in paediatric populations in a community setting were identified (Table 3). Studies from Netherlands [21], the US [22], UK [11], Germany [23], and Denmark [24] were included.

**Table 3:** Characteristics of studies included for comparative data and comparison of average prescribing rates per 1000 GMS children between 2001 and 2011, to European rates per 1000 children

Study (publication year)	Country (year data represents)	Sample Size	Age	Overall Rate of Anti-depressant Prescribing (per 1000)	Setting
Dorks (2013) [23]	Germany (2004–2006)	2 599 685	0–17	1.7/1000	Pharmaco-epidemiology database
Volkers (2007) [24]	Netherlands (2001–2005)	350 000	0–17	2.2/1000	Information Network of General Practice
Wijlaars (2012) [12]	UK (1995–2009)	1 502 753	0–17	3.6/1000	UK primary care database
Parkinson (2012) [25]	US (2002–2005)	32 111	0–17	8.77/1000	Medical Expenditure Panel survey
Steinhausen (2014) [26]	Denmark (1996–2010)	105 908	0–17	1.6/1000	National Patient Database
GMS data	Ireland (2002 – 2011)	311 579	0–15	3.3/1000	Primary care reimbursement service pharmacy claims

The overall prevalence of anti-depressants for the study period was 3.3/1000 population. Only one country reported a higher overall prevalence, the US study conducted between 2002 and 2005 (8.77/1000 population) [22]. Table 3 shows that the prevalence of anti-depressants in paediatrics varies substantially with Ireland ranked higher than the majority of comparison countries. However, there are differences across the different studies in terms of age groups, sample size and year the data were assessed.

## Discussion

The study observed that SSRI prescription rates reduced significantly following the introduction of the IMB warning. It showed that paroxetine rates reduced from 2002 onwards, which may indicate the influence of media coverage on Irish prescribing. Results show that adolescent girls are more likely to receive a prescription of anti-depressants than adolescent boys and the overall prevalence of anti-depressants in 0–15 year olds in Ireland lies close to the median value in comparison to international studies.

An overall reduction in the prevalence of SSRIs and all anti-depressants in children over the study period was seen. These reductions appear to be influenced by the IMB and MHRA regulatory recommendations of 2003. Higher rates of fluoxetine prescribing further supports this. The MHRA deemed fluoxetine an acceptable treatment of MDD in children, a stance which was adopted by the IMB. Interestingly, the prevalence of fluoxetine increases from 2002 to 2011. This suggests that while the prevalence of SSRIs decreased over time, the prevalence of fluoxetine, in Ireland, was not affected by the general black box warning placed on all SSRIs by the FDA.

In addition to an overall reduction in the prevalence of anti-depressants, the study observed a significant reduction in the prevalence of contra-indicated anti-depressants - paroxetine and citralopram and to some extent sertraline. This decrease, coupled with the increase of fluoxetine prescribing, suggests a possible link between the IMB warning and prescription practices. The decrease in citralopram began in 2003, the year the warning was issued. The decline in paroxetine use was more pronounced than the other SSRIs and began in 2002, prior to the IMB warnings [25].

Since 2005 the prevalence of SSRIs in children on the GMS has remained relatively stable (2.1/1000 population). While not significant, the prevalence increased in the latter part of the study period. This may indicate that concerns about the risks associated with SSRIs have dissipated in recent years. This may be due to contradictions that exist in recent literature regarding the safety and efficacy of SSRIs. For example, some studies report no increased risk of suicidality following SSRI prescriptions in childhood [26] and others suggest that a relationship exists between decreases in the prevalence of SSRIs and increases in suicide behaviour among young people [27]. Contrary to this view a recent review of published and unpublished SSRI research revealed significant increases in suicidal behaviour in children taking contraindicated SSRIs [12–14, 28, 29].

Age and gender analysis revealed that before adolescence (<12 years of age), boys were more likely to be prescribed an anti-depressant than girls. This trend reversed after age 12 where significantly more girls received an anti-depressant prescription than boys. This finding is in line with previous research, whereby prepubescent boys show higher rates of anti-depressant prescriptions than prepubescent girls [11], and adolescent girls show higher rates of anti-depressant prescriptions than adolescent boys [11, 21, 24]. Research shows that girls aged 3–13 years are less likely than boys to be diagnosed with major depression and girls age 12–17 are more likely to meet the diagnostic criteria of major depression than boys [11]. Age trends reveal that older children (12–15 age groups) are more likely to be prescribed an anti-depressant than both younger age groups. This is consistent with previous research which shows that the rate of anti-depressant and overall psychotropic prescriptions increases with age [18, 27].

The prevalence of anti-depressants in the current study are similar in size and trend to those found in the UK primary care database which examined prevalence from 1995 to 2009 [11]. They found a reduction of SSRI rates following the FDA warning. However, of the contra-indicated SSRIs only paroxetine showed a significant decrease following 2003; citralopram and sertraline were not affected. This difference in contra-indicated prescription trends may be a consequence of methodological differences between our study and the UK study. Our study examined year on year prescription rates, whereas the UK study looked at 3 time periods (1995 – 2002, 2003–2005, and 2006–2009).

While the overall prevalence for the current study was similar to the UK, it was higher than Germany, the Netherlands and Denmark. Denmark showed the smallest overall prevalence and the Danish prescribing trends did not seem to be affected by the FDA warning. The rates in the US study were twice the rate of our study, which is in line with previous research findings [17]. The differences in prescription trends may be due to high levels of heterogeneity between the studies, cultural variation in prescription practices and differences in the availability of other treatments options [28].

### Limitations

The HSE-PCRS GMS pharmacy claims database represents approximately one-third of Irish children and over-represents more socially disadvantaged children in the Irish population. This may result in an over-estimation of the true trends in the prevalence of anti-depressants, given that children from lower socioeconomic backgrounds are more likely to be prescribed a psychotropic medication [29, 30] and are at greater risk of depression [11] and anxiety related disorders [31]. Direct comparison of international prevalence for low socioeconomic populations was limited; however studies that have included deprivation as an indicator of prevalence show that as deprivation increases the prevalence of anti-depressants increases also [11].

The GMS data set does not collect information about the indication for prescriptions or about the setting in which the prescription was initiated (e.g. primary care, hospital or specialist setting). In addition, there are no national rates of childhood depression available in Ireland to compare current prescription rates to. This lack of diagnostic information makes it difficult to know whether the current rates reflect treatment of depression, or other conditions (obsessive compulsive disorder (OCD) or anxiety). We know that cultural differences exist in terms of what indications anti-depressants are prescribed for. For example, in the US they are often prescribed for depression and ADHD, whereas in Europe OCD and depression are most likely to receive an anti-depressant prescription [17]. The current data does not allow us to explore the indications for which anti-depressants are most commonly prescribed in Irish children. Furthermore, there is no data on dispensing and treatment compliance; hence the current rate may not accurately reflect actual anti-depressant use.

## Conclusions

After 2003, a significant decrease in the prevalence of SSRIs in children, particularly paroxetine and citralopram, was found. The prevalence of fluoxetine remained stable and increased from 2002 to 2011. The direction and timing of these trends suggest that medical practitioners followed the FDA and CSM advice, although the earlier reduction of paroxetine would suggest that the negative media attention had an influence on prevalence, though it is unknown whether this effect was patient or practitioner driven.

## Abbreviations

GMS: General Medical Services
SSRIs: Selective serotonin reuptake inhibitors
RCT: Randomised controlled trials
FDA: Food and Drug Administration
MDD: Major depressive disorder
MHRA: Medicines and Health Regulatory Agency
IMB: Irish Medical Board
ATC: Anatomical Therapeutic Chemical
